# Studies on the antidiabetic activities of *Momordica charantia* fruit juice in streptozotocin-induced diabetic rats

**DOI:** 10.1080/13880209.2016.1275026

**Published:** 2017-01-09

**Authors:** Mona F. Mahmoud, Fatma El Zahraa Z. El Ashry, Nabila N. El Maraghy, Ahmed Fahmy

**Affiliations:** a Department of Pharmacology and Toxicology, Faculty of Pharmacy, Zagazig University, Zagazig, Egypt;; b Department of Pharmacology and Toxicology, Faculty of Pharmacy, Future University, Future, Egypt

**Keywords:** Diabetes, insulin resistance, diaphragm

## Abstract

**Context:***Momordica charantia* Linn (Cucurbitaceae) (MC) is used in folk medicine to treat various diseases including diabetes mellitus.

**Objective:** This study investigates the antidiabetic activities of *Momordica charantia* (bitter gourd) on streptozotocin-induced type 2 diabetes mellitus in rats.

**Materials and methods**: Male Wister rats were randomly assigned to 4 groups. Group I, Normal control; Group II, STZ diabetic; Group III and IV, *Momordica charantia* fruit juice was orally administered to diabetic rats (10 mL/kg/day either as prophylaxis for 14 days before induction of diabetes then 21 days treatment, or as treatment given for 21 days after induction of diabetes). The effects of MC juice were studied both *in vivo* and *in vitro* by studying the glucose uptake of isolated rat diaphragm muscles in the presence and absence of insulin. Histopathological examination of pancreas was also performed.

**Results**: This study showed that MC caused a significant reduction of serum glucose (135.99 ± 6.27 and 149.79 ± 1.90 vs. 253.40* ± 8.18) for prophylaxis and treatment respectively, fructosamine (0.99 ± 0.01 and 1.01 ± 0.04 vs. 3.04 ± 0.07), total cholesterol, triglycerides levels, insulin resistance index (1.13 ± 0.08 and 1.19 ± 0.05 vs. 1.48 ± 1.47) and pancreatic malondialdehyde content (*p* < 0.05). While it induced a significant increase of serum insulin (3.41 ± 0.08 and 3.28 ± 0.08 vs. 2.39 ± 0.27), HDL-cholesterol, total antioxidant capacity levels, β cell function percent, and pancreatic reduced glutathione (GSH) content (*p* < 0.05) and improved histopathological changes of the pancreas. It also increased glucose uptake by diaphragms of normal (12.17 ± 0.60 vs. 9.07 ± 0.66) and diabetic rats (8.37 ± 0.28 vs. 4.29 ± 0.51) in the absence and presence of insulin (*p* < 0.05).

**Conclusions:***Momordica charantia* presents excellent antidiabetic and antioxidant activities and thus has great potential as a new source for diabetes treatment whether it is used for prophylaxis or treatment.

## Introduction

The prevalence of diabetes is increasing at an alarming rate and has become one of the world's most serious public health problems. Diabetes is also considered the most common endocrine disorder. The American Diabetes Association (ADA) showed that diabetes is the fourth leading cause of death in the USA. Medical and other related costs can so far over $100 billion a year; 380 million people are expected to suffer from the disease by 2025, with cases being found increasingly in developing countries (Palaian et al. 2005; Center for Disease Control and prevention 2007).

Bitter gourd (Bitter melon) or *Momordica charantia* Linn. (Cucurbitaceae) (MC) is a tropical and subtropical vine (Garau et al. 2003). It is used in the Ayurvedic system of medicine for treating various diseases including diabetes mellitus (Yadav et al. 2005). In addition to its major use as an antidiabetic agent, MC has been used in India and Sri Lanka as a tonic, emetic and laxative. Both the cultivated and wild forms are used for this purpose (Karunanayake & Tennekoon 1993). The major compounds that have been isolated from MC and identified as hypoglycaemic agents include charantin, vicine, polypeptide-p or p-insulin and kakra compounds (three non-steroidal hypoglycaemic compounds isolated from the fruit) (Srivastava et al. 1993).

Some compounds of MC have been reported to treat and prevent diabetic symptoms by mechanisms other than lowering blood glucose. For instance, many of the antioxidants found in MC work by protecting the body’s cells from oxidative damage. One study discovered that conjugated linolenic acid which is one of major antioxidant involved in preventing type-2 diabetes occurs abundantly (57.7%) in the seed oil of MC (Dhar et al. 2006). Another mechanism whereby MC bioactive compounds indirectly lowers blood glucose by reducing adiposity and normalizing glucose tolerance. Researchers have discovered that the bioactive compounds in MC have hypolipidemic actions that can lower serum and liver cholesterol, which improves glucose tolerance (Matsui et al. 2013). Insulinomimetic proteins (galactose binding lecithin) with a molecular weight of 124,000 have been isolated from MC. It has antilipolytic and lipogenic activities in isolated rat adipocytes similar to insulin, (Chen et al. 2003). It has also free radical scavenging activity and protects against lipid peroxidation (Chaturvedi 2009).

There is little information regarding enteral feeding of this natural product as functional food to improve glycaemic condition in diabetes. This study investigates the effects of orally administered MC by using STZ-induced diabetic rat model. Furthermore, *in vitro* glucose uptake by isolated rat diaphragm was estimated to investigate the probable underlying mechanisms of action of this natural agent. In our present study, we also compared the prophylactic and the curative effects of MC.

## Materials and methods

### *In vivo* studies

#### Drugs and chemicals

STZ was supplied by Sigma Chemicals St. Louis, MO. Heparin sodium ampoule from El-Nile Pharmaceuticals Company, Cairo, Egypt and potassium phosphate from El-Nasr Pharmaceutical Chemicals Co., Cairo, Egypt. *Momordica charantia* was obtained from local market, in August, September, October and November 2012 in Egypt. It was identified by Prof. Dr Assem El Shazly, Department of Pharmacognosy, Faculty of Pharmacy, Zagazig University. A voucher specimen was kept in Pharmacognosy Department herbarium.

#### Preparation of fruit extract

*Momordica charantia* fruit juice was prepared as described by Sharma et al. (1995). Briefly, fresh fruit (1 kg) was washed thoroughly. The juice was obtained by using a commercial juice extractor (Moulinex, France). The fresh juice was centrifuged at 5000 rpm for 30 min and the clear supernatant was considered as 100%. *Momordica charantia* fruit juice was diluted with autoclaved distilled water to make 50% juice according to Sitasawad et al. (2000). It was stored at 4 °C and administered by oral gavage daily at a dosage of 10 mL/kg body weight.

#### Animals

Adult male albino rats (aged 6–8 weeks) weighing 150–200 g (National Research Center Laboratory, Cairo, Egypt) were housed in standard polypropylene cages (three rats per cage) and kept on a light-dark cycle of equal duration, under constant environmental conditions. Rats were fed commercially available rat normal pellet diet (carbohydrates 35%, proteins 25%, lipids 7% and vitamins 3%) and water *ad libitum*. Animal use protocols employed in this study were approved by Ethical Committee of the Faculty of Pharmacy, Zagazig University for Animal Use and conducted in accordance with the guidelines of the US National Institutes of Health on animal care.

#### Induction of type-2 diabetes

After an overnight fasting, hyperglycaemia and overt diabetes were induced by an intraperitoneal injection of a single dose of freshly prepared STZ (45 mg/kg) in citrate buffer (0.09 M, pH 4.8) (Tikkanen et al. 1998). Normal control rats received intraperitoneal citrate buffer only. Animals were monitored by periodic estimation of body weight and biochemical testing of fasting serum glucose, and only those with persistent blood glucose levels ≥200 mg/dL, for 7 days after STZ administration, were considered diabetic and included in the study.

#### Experimental design

Animals were randomly assigned to four groups (*n* = 6–8). Group I, Normal control rats; Group II, STZ diabetic rats; Group III, rats pretreated with the *Momordica charantia* (10 mL/kg) for 14 days before induction of diabetes then 21 days treatment. Group IV. Diabetic rats received *Momordica charantia* (10 mL/kg) daily for 21 days.

#### Blood sampling and serum separation

At the end of the study period, blood samples were collected from the retro-orbital venous plexus of ether anesthetized rats (Schemer 1967) in dry glass centrifuge tubes and centrifuged at 3700 rpm for 20 min at room temperature. Serum was separated and 10 μL was immediately used for determination of blood glucose level and the remaining amount of serum was stored at −20 °C for determination of other parameters.

#### Tissue sampling

The animals were divided into two sets. The first set was used to prepare tissue homogenates as follows: prior to dissection, pancreatic tissues were perfused with a phosphate buffered saline solution, pH 7.4 containing 0.16 mg/mL heparin to remove any red blood cells and clots. After dissection of pancreas, the adherent lipid parts were removed and each pancreas was dried, weighed then homogenized in 5 mL cold buffer (50 mM potassium phosphate, PH 7.5) per gram tissue. The homogenates were centrifuged at 4000 rpm for 15 min and the supernatants were removed and frozen at −80 °C for determination of pancreatic malondialdehyde (MDA) and reduced glutathione (GSH) contents. The second set of animals were sacrificed and dissected. Tissue samples from pancreas were removed and fixed in 10% formalin in saline solution and processed for histopathological examination.

#### Biochemical analyses

Serum glucose level, total cholesterol, triglycerides and HDL-cholesterol levels were measured enzymatically using kits purchased from Spinreact Co., Spain. Serum insulin level was determined by radioimmunoassay method using a kit purchased from Diagnostic Products Co. (DPC), LA (Feldman & Roadbard 1971). Serum fructosamine level was determined by enzymatic colorimetric method using Quimica (QCA) kit (Amposta, Spain) (Armbruster 1987). Total antioxidant capacity (TAOC) was measured in serum using kit purchased from Bio-diagnostic Co., Egypt. The determination of antioxidant capacity was performed by the reaction of antioxidants in the sample with a defined amount of exogenously provided hydrogen peroxide (H_2_O_2_). The antioxidants in the sample eliminate a certain amount of the provided hydrogen peroxide. The residual H_2_O_2_ was determined colorimetrically by an enzymatic reaction which involves the conversion of 3,5-dichloro-2-hydroxy benzene sulfonate to a coloured product (Koracevic et al. 2001).

Pancreatic MDA content was determined by the thiobarbituric acid (TBA) method using a kit purchased from Bio-diagnostic Co., Egypt (Ohkawa et al. 1979). Pancreatic reduced glutathione content was also determined colorimetrically (Beutler et al. 1963). Both insulin resistance and β cell function were calculated by Using HOMA-IR calculator version 0.3 (Homeostasis Model Assessment ‘HOMA’, which is a computer-solved model of insulin and glucose interactions) (Matthews et al. 1985).

#### Histopathological examination

The pancreatic tissues were fixed by immersion at room temperature in 10% neutral formalin solution. Sections of 5–6 μm thickness were stained with haematoxylin and eosin (H & E) then examined under light microscope for determination of histopathological changes. The histological analysis was performed by a person blinded to the treatment.

### *In vitro* studies

Adult male albino rats (8 weeks old), weighing 200–250 g, were used in the present part of the study to investigate the effects of *Momordica charantia* on glucose uptake by diaphragms isolated from normal and STZ diabetic rats in presence or in absence of insulin according to the method described by Ghosh et al. (2004) with some modification. The chosen weights of animals were based on the fact that younger animals had too small diaphragms to yield the desired number of samples, while, larger animals (more than 300 g) had too thick diaphragms to allow complete oxygen diffusion. Rats were fasted overnight and killed. The diaphragms were dissected out quickly with minimal trauma and divided into four parts with equal weights.

Two main groups of normal rats diaphragms or STZ diabetic rats diaphragms, each of them was subdivided into four subgroups containing six numbers of graduated test tubes (*n* = 6) each containing 2 mL of Tyrode’s solution and 3 g/L glucose. Subgroup 1 has 2 mL Tyrode’s solution containing 3 g/L glucose only. Subgroup 2 contains also regular insulin (Novo Nordisk, Cairo, Egypt.) 0.4 mL of 100 units/mL solution. Subgroup 3 contains 2 mL of Tyrode's solution containing 3 g/L glucose and 0.5% MC fruit juice (0.02 mL) according to Sitasawad et al. (2000).

Subgroup 4 includes regular insulin 0.62 mL of 0.4 units per mL solution and 0.5% MC fruit juice (0.02 mL). The volumes of all the test tubes were made up to 4 mL with distilled water. Incubation of the diaphragms was performed for 30 min in shaking water path at 37 °C with carbogen gas (95% O_2_ and 5% CO_2_) flow through the incubation tubes. Glucose concentration was measured before and after incubation using the previously mentioned enzymatic method. Glucose uptake per gram of tissue was calculated as the difference between the initial and final glucose content in the incubated medium. Tyrode’s solution consists of 8 g of NaCl, 0.2 g of KCl, 0.2 g of CaCl_2_, 0.1 g of MgCl_2_.6H_2_O, 0.05 g of NaH_2_PO_4_, 1 g of NaHCO_3_. Then, 3 g of d-glucose is added, and water to make 1000 mL

### Statistical analysis

All data are expressed as mean ± SEM. Statistical analysis was performed by the analysis of variance (ANOVA) followed by Bonferroni *post hoc* test and Student’s *t-*test for unpaired data (Snedecor & Cochran 1967) using Graph Pad prism program version 5. A value of *p* < 0.05 was used as the limit for statistical significance.

## Results

### *In vivo* study

#### Biochemical and histopathological changes in diabetic group

**Figure 1.**
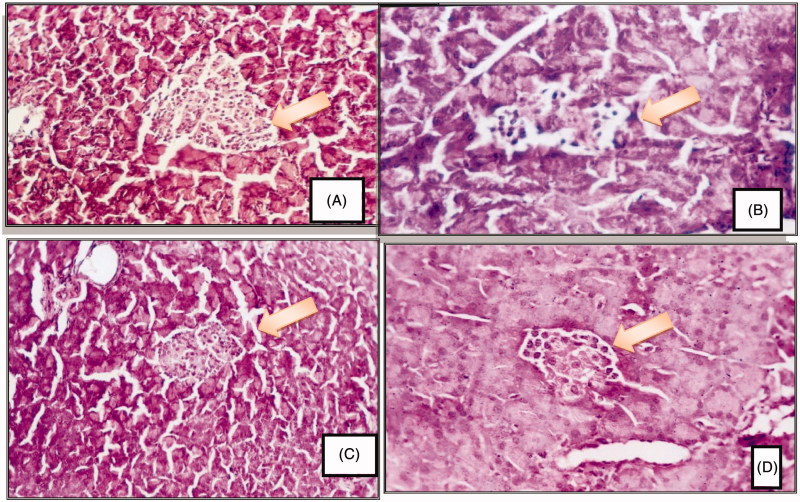
Photomicrographs are representative of cross sections from six rats stained with hematoxylin and eosin. (A) showing pancreas of normal rat with normal pancreatic tissue including islets of Langerhans, pancreatic acini and ducts, (B) showing pancreas of diabetic rat with islet destruction, reduction in size and lymphocytic infiltration, (C) representing pancreas of *Momordica charantia* pretreated diabetic rat showing mild islets destruction, (D) showing pancreas of diabetic rat treated with *Momordica charantia* with moderate islet destruction (H&E 250×).

STZ induced a significant increase in serum glucose (253.40 ± 8.18 vs. 82.59 ± 3.82), fructosamine (3.04 ± 0.07 vs. 0.96 ± 0.04), TC, TG levels, insulin resistance index (1.48 ± 1.47 vs. 0.94 ± 0.22) and pancreatic MDA content in comparison to normal control (*p* < 0.05). There was also a significant decrease in serum insulin (2.39 ± 0.27 vs. 4.67 ± 0.22), serum HDL-c, serum TAOC levels, β cell function percent (4.34 ± 0.20 vs. 87.41 ± 3.44) and pancreatic GSH content (*p* < 0.05) as seen in Figure 3. Histopathological examination of pancreas revealed severe islet destruction, reduction of islet size, congestion of blood vessels, lymphocytic infiltration and vascular degenerative changes in the islets as shown in Figure 1(B).

#### Biochemical and histopathological changes in treated diabetic groups

**Figure 2.**
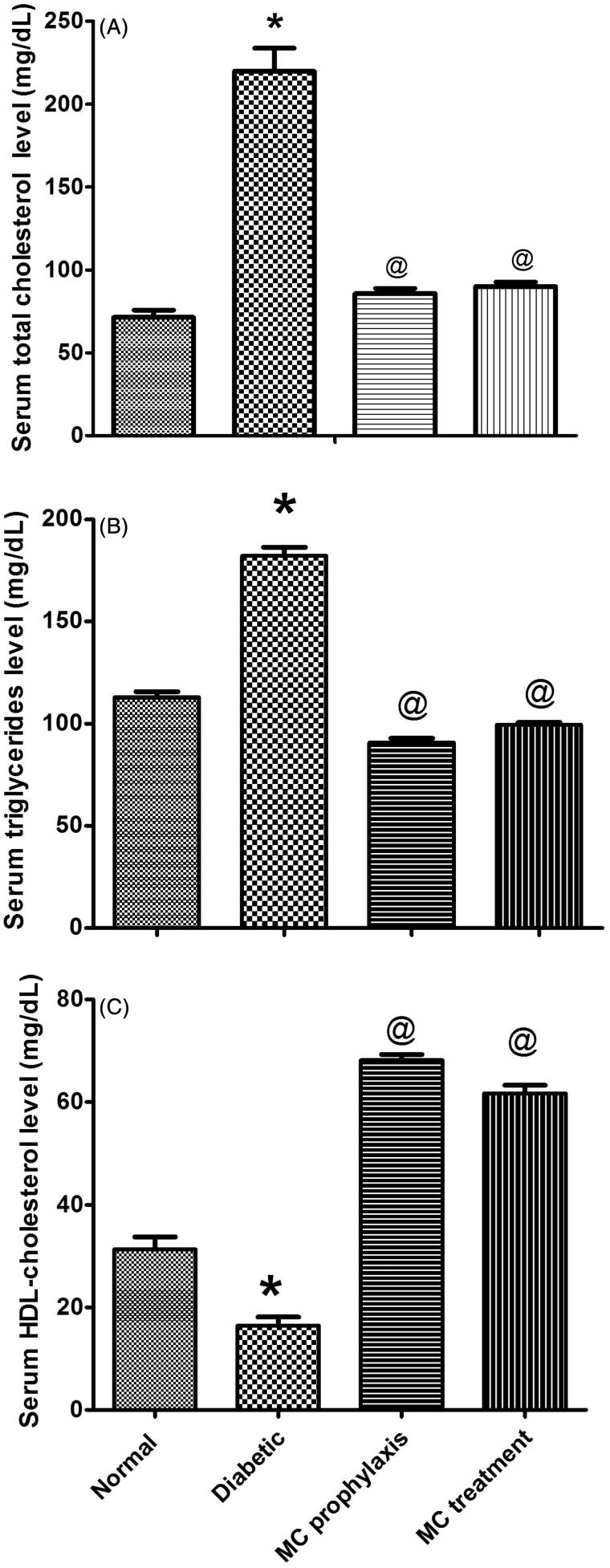
Effect of oral administration of 50% *Momordica charantia* (MC) fruit juice (10 mL/kg) on serum cholesterol (A), triglycerides (B) and serum HDL-C (C), in pretreated and treated diabetic rats. (*n* = 6), *p < 0.05 (significantly different from normal group); #*p* < 0.05 (significantly different from diabetic group) by one-way ANOVA and Bonferroni *post hoc* test.

**Figure 3.**
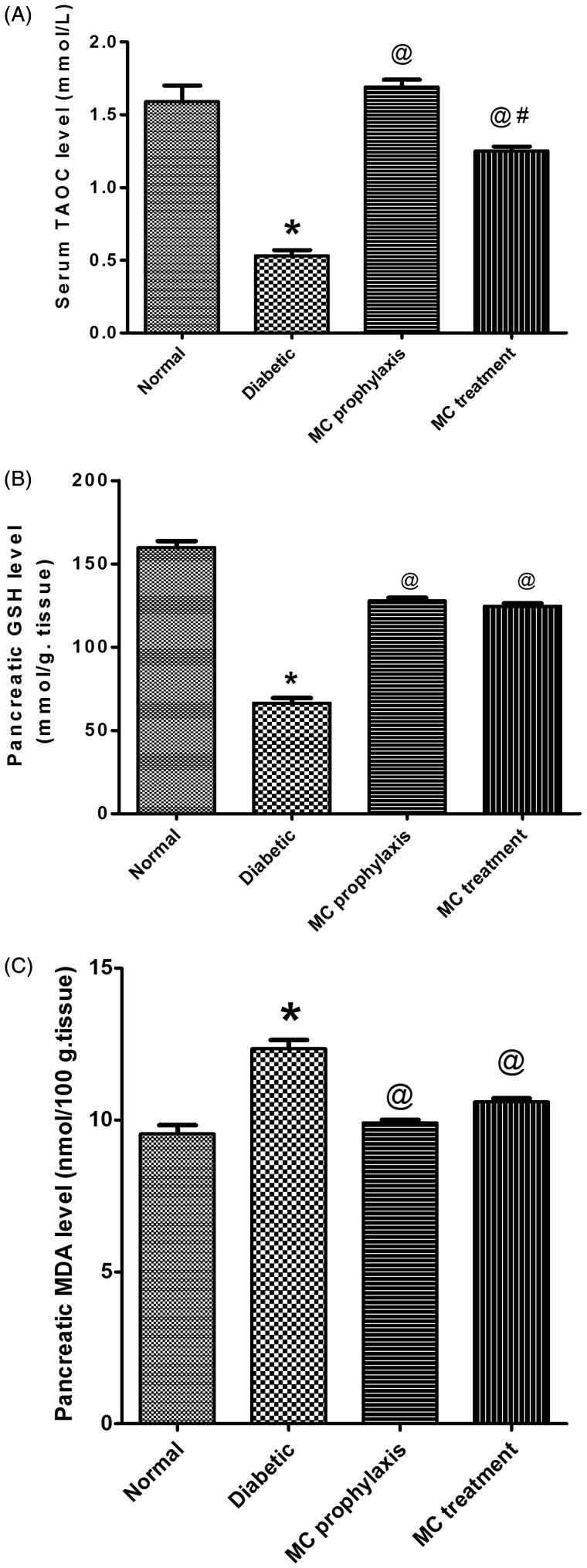
Effect of oral administration of 50% *Momordica charantia* (MC) fruit juice (10 mL/kg) on serum total antioxidant capacity (TOAC) (A), pancreatic reduced glutathione (GSH) (B) and pancreatic MDA (C), in pretreated and treated diabetic rats. (*n* = 6), *p < 0.05 (significantly different from normal group); #*p* < 0.05 (significantly different from diabetic group) by one-way ANOVA and Bonferroni *post hoc* test.

As shown in Table 1, the diabetic groups treated with *Momordica charantia* showed a significant decrease in serum glucose (135.99 ± 6.27 and 149.79 ± 1.90 vs. 253.40 ± 8.18) for prophylaxis and treatment respectively, fructosamine levels (0.99 ± 0.01 and 1.01 ± 0.04 vs. 3.04 ± 0.07)and in insulin resistance index (1.13 ± 0.08 and 1.19 ± 0.05 vs. 1.48 ± 1.47) while serum insulin levels (3.41 ± 0.08 and 3.28 ± 0.08 vs. 2.39 ± 0.27), and β-cell function percent (17.63 ± 0.61 and 13.85 ± 0.30 vs. 4.34 ± 0.20) were significantly increased (*p* < 0.05). Figure 2 showed significant lowering in TC, TG and increasing HDL-c levels. Meanwhile, MC significantly increased TAOC levels and decreased pancreatic MDA content. However, pancreatic GSH content was significantly increased (*p* < 0.05) (Figure 3). There was no significant difference between prophylaxis and treatment groups in all studied parameters (*p* > 0.05). Histopathological examination of pancreas isolated from prophylaxis group (Figure 1(C)) and treatment groups (Figure 1(D)) showed milder islet destruction with little mononuclear cell infiltration when compared with diabetic control. However, the effect of MC when used as prophylaxis was better on histopathological changes than when used as treatment.

**Table 1.** Effect of oral administration of 50% *Momordicacharantia* (MC)fruit juice (10 ml/kg) on serum glucose, insulin, insulin resistance index, β-cell function percent and serum fructosamine level, in diabetic rats.

Parameters	Normal	Diabetic	MC Prophylaxis	MC treatment
Glucose (mg/dL)	82.59 ± 3.82	253.40* ± 8.18	135.99^#^ ± 6.27	149.79^#^ ± 1.90
Insulin (μIU/mL)	4.67 ± 0.22	2.39* ± 0.27	3.41^#^ ± 0.08	3.28^#^ ± 0.08
Insulin resistance	0.94 ± 0.22	1.48* ± 1.47	1.13^#^ ± 0.08	1.19^#^ ± 0.05
β-Cell function (%)	87.41 ± 3.44	4.34* ± 0.20	17.63^#^ ± 0.61	13.85^#^ ± 0.30
Fructosamine (mmol/L)	0.96 ± 0.04	3.04* ± 0.07	0.99^#^ ± 0.01	1.01^#^ ± 0.04

Results are presented as mean ± S.E.M (*n* = 6).

* *p* < 0.05 (significantly different from normal group); #*p* < 0.05 (significantly different from diabetic group) by one-way ANOVA and Bonferroni *post hoc* test.

### *In vitro* results

*In vitro* study showed that STZ induced a significant reduction in glucose uptake by isolated rat diaphragms from the physiological solution (2.54 ± 0.47 vs. 4.72 ± 0.44) in the absence of insulin (*p* < 0.05) and (4.29 ± 0.51 vs. 9.07 ± 0.66) in the presence of insulin (Table 2a). At the same time, it is clear (Tables 2a and 2b) that MC was able to increase glucose uptake by diaphragms isolated from normal (6.03 ± 0.20 vs. 4.72 ± 0.44 in the absence of insulin and 12.17 ± 0.60 vs. 9.07 ± 0.66 in the presence of insulin) and from diabetic rats in absence (3.94 ± 0.26 vs. 2.54 ± 0.47) and in presence of insulin (8.37 ± 0.28 vs. 4.29 ± 0.51) (*p* < 0.05).

**Table 2a.** Effect of 0.5% *Momordica charantia* (MC) fruit juice (0.02 ml) and STZ on glucose uptake of diaphragms isolated from normal rats in absence and in presence of insulin.

Diaphragms	Glucose uptake (mg/g tissue/30 min)			
	In absence of insulin		In presence of insulin	
	Mean ± S.E	%Effect	Mean ± S.E	%Effect
Normal	4.72 ± 0.44	100.00	9.07*± 0.66	100.00
STZ	2.54* ± 0.47	53.81	4.29*± 0.51	47.30
MC	6.03*^@^ ± 0.20	127.75	12.17*^@^ ± 0.60	134.18

Data are presented as mean ± S.E (*n* = 6).

* Significantly different from normal control at *p* < 0.05.

@ Significantly different from diabetic rats at *p* < 0.05.

**Table 2b.** Effect of 0.5% *Momordica charantia* (MC) fruit juice (0.02 ml) on glucose uptake of diaphragms isolated from diabetic rats in absence and in presence of insulin.

Diaphragms	Glucose uptake (mg/g tissue/30 min)			
	In absence of insulin		In absence of insulin	
	Mean ± S.E	%Effect	Mean ± S.E	%Effect
Diabetic	2.54 ± 0.47	100.00	4.29 ± 0.51	100.00
MC	3.94*± 0.26	155.12	8.37*^@^ ± 0.28	195.10

Data are presented as mean ± S.E (*n* = 6).

* Significantly different from diabetic control at *p* < 0.05.

@ Significantly different from diabetic rats + insulin at *p* < 0.05.

## Discussion

The body’s response to blood sugar requires the coordination of an array of mechanisms. Failure of any of the components involved in insulin regulation, secretion, uptake or breakdown can lead to the build-up of glucose in blood. Likewise, any damage to the β cells, which produce insulin, will lead to persistent hyperglycaemia, the common characteristics of diabetes (Wahren Ekberg 2007).

Different approaches have been used to reduce the incidence of diabetes. Although, oral hypoglycemic agents and insulin are the mainstays of treatment of diabetes and are effective in controlling hyperglycaemia, they have prominent side effects and fail to significantly alter the course of diabetic complications (Rang & Dale 1991). Dietary strategies should aim to normalize blood glucose and lipoprotein levels in order to reduce morbidity and mortality related to derangement of carbohydrate and lipoprotein metabolism in DM.

One plant that has received the most attention for its antidiabetic properties is bitter melon, *Momordica charantia*, commonly referred to as bitter gourd (b.g), karela or balsam pear (Yibchok-Anun et al. 2006). Results of this investigation suggested that MC fruit juice caused a significant reduction in serum glucose with a significant elevation of serum insulin levels in diabetic and pretreated rats. It also increased β-cell function percent in both groups. This may be due to its pancreatic and/or extra-pancreatic effects.

These findings are in agreement with that reported by Rotshteyn and Zito (2004) and Fernandes et al. (2007) who found that MC extract administration to alloxan diabetic rats was able to reduce blood glucose and elevate the reduced serum insulin similar to glibenclamide administration. It was also assumed that it might stimulate the secretion of insulin from β cells by a mechanism similar to that of oral hypoglycemic agents (like sulfonylurea) i.e., by depolarization of β cells membrane which consequently alters ion flux (Grodsky et al. 1977) or affecting receptors responsible for the recognition of insulin secretagogues (Hellman et al. 1973). These mechanism(s) have been accepted as a paradigm for the action of all insulin releasing agents (Fernandes et al. 2007).

The possible mechanism by which MC extract brings about a decrease in blood glucose may be via stimulation of surviving β cells to release more insulin (Sathishsekar & Subramanian, 2005). This was clearly evidenced by the increased level of plasma insulin in diabetic rats treated with MC extract. The activation of β cells with MC extract was reported in mildly STZ diabetic animals in which some β cells were found active and granulation returns to normal giving insulinogenic effect.

It has been hypothesized that MC extract increases β cell proliferation in the pancreas; however, this mechanism has not been confirmed by studies (Day et al. 1990; Sarkar et al. 1996). Previous studies demonstrated that MC increase the number of β cells in the pancreas thereby improving the body's ability to produce insulin (Kumar et al. 2010).

It was demonstrated that components of MC extract appear to have structural similarities to animal insulin, as measured by electrophoresis and infra-red spectrum analysis (Wong et al. 1985; Ng et al. 1986). It was demonstrated that MC contains a lecithin that has insulin-like activity. This lecithin lowers blood glucose concentrations by acting on peripheral tissues and, similar to insulin's effects in the brain, suppressing appetite. This lecithin is likely a major contributor to the hypoglycemic effect that develops after eating MC (Kumar et al. 2010).

Histopathological examination of pancreas of diabetic rats treated with MC juice showed a marked pancreatic islets destruction and reduction in size, but the pancreatic damage observed in this group was milder than that found in the untreated diabetic control group. The pretreatment by MC juice before induction of diabetes produced only mild islet cells destruction with a less marked cellular degeneration of pancreatic β cells. The more potent effect of MC pretreatment on structural changes is attributed to the more potent antioxidant effect. MC pretreatment exerted more potent increase in the total antioxidant capacity and more reduction of lipid peroxidation compared with MC treatment. These results are in harmony with the findings of Fernandes et al. (2007) who reported that MC extract administration to alloxan diabetic rats was able to significantly improve the histological architecture of the islets of Langerhans. Oral feeding of fruit juice may have a role in the renewal of β cells in STZ-diabetic rats or may permit the recovery of partially destroyed β cells (Ahmed et al. 1998).

There is evidence indicating that MC may decrease hepatic gluconeogenesis, increase hepatic glycogen synthesis, and increase peripheral glucose oxidation in erythrocytes and adipocytes (Shibib et al. 1993). For further screening of the mechanism(s) by which MC exerts its hypoglycemic action, our *in vitro* studies indicated that MC juice can stimulate glucose uptake by diaphragms taken from normal and diabetic rats. Administration of MC and insulin together was found to be more effective than each alone. This may be due to potentiation of insulin action and increasing tissue sensitivity to insulin. These results correspond with the findings of Fernandes et al. (2007) and Kumar et al. (2010). Oral MC treatment increased muscle content of facilitative glucose transporter-4 (GLUT-4) protein in the plasma membrane fraction from the muscle (Miura et al. 2001). This may be responsible for amelioration of insulin resistance in diabetes.

This study also demonstrated an increase in the antioxidant capacity with a decrease in the production of reactive oxygen species. MC fruit extract contains low-molecular-weight antioxidant compounds, such as polyphenols, flavonoids and flavonols. It has been reported that MC extract increases glucose uptake by augmenting phosphatidyl inositol-3-kinase pathway (Tiwari 2007). Many reports suggested that MC possesses insulin-like activity responsible for augmentation of glucose as well as amino acid uptake into skeletal muscle cells (Ahmed et al. 2004; Cummings et al. 2004). The antidiabetic properties of MC are attributed to charantin, the key constituent of *M*. *charantia* (Joseph & Jini 2013).

The decrease in glucose level and increase in insulin level may decrease protein glycation. This was observed in this study, where MC fruit juice reduced serum fructosamine levels in diabetic and in MC-pretreated rats. This effect is in full agreement with that reported by Petlevski et al. (2000).

High consumption of vegetables and fruits is associated with lowering plasma lipids (Koebnick et al. 2005). This study demonstrated that MC fruit juice also possesses lipid lowering properties in diabetic animals; 2- or 4-fold increase in serum TC and serum TG levels was observed in STZ-induced diabetic rats, but in MC treated and pretreated diabetic rats, these levels were significantly reduced with a significant elevation in serum HDL-c levels. This hypolipidemic effect may possibly mediated by controlling the hydrolysis of certain lipoproteins and their selective uptake and metabolism by different tissues.

The anti-hyperlipidemic effect of MC may be due to the down regulation of NADPH and NADH cofactors in the fat metabolism (Fernandes et al. 2007). MC may exert its anti-hyperlipidemic action by oxidizing NADPH. Furthermore, insulin inhibits adipose tissue hormone-sensitive lipase and, therefore, reduces lipolysis and mobilization of peripheral depots. MC fruit may mimic the action of insulin or may have a synergistic effect on insulin activity. It was demonstrated that MC reduces cellular TG synthesis and secretion as well as apolipoprotein B secretion (Nerurkar 2005). MC juice reduces adiposity in rats fed a high-fat diet (Virdi et al. 2003).These observations provide evidence that MC may be beneficial in preventing or delaying the development of cardiovascular complications in diabetics.

This study indicated that MC fruit juice possesses potent antioxidant activity, which may be directly or indirectly responsible for its hypoglycemic property. In this study, MC significantly lowered pancreatic MDA in diabetic and pretreatment group. This may be due to reducing the rate of lipid peroxidation (Sitasawad et al. 2000) and its cytoprotective action (Ahmed et al. 1998).

MC also produced a significant increase in serum TAOC level and on pancreatic GSH level in both diabetic and MC pretreatment groups. This can be due to either increase the biosynthesis of GSH and other antioxidant enzymes in the enzymatic antioxidant defense system or reduce the oxidative stress leading to less degradation of them, or have both effects. The extract exerted rapid protective effects against lipid peroxidation by scavenging free radicals there and reducing the risk of diabetic complications. MC fruit juice was found to play a role in reducing levels of lipid peroxidation *in vivo* as well as *in vitro* models (Sitasawad et al. 2000).

In conclusion, this study demonstrated that MC showed hypoglycemic, hypolipidemic and strong antioxidant properties when orally administrated as either prophylaxis or treatment to STZ diabetic rats. It was able to reverse STZ pathological features by controlling hyperglycaemia, hyperlipidaemia and oxidative stress when used as a pretreatment before induction of DM. However, it was unable to prevent the occurrence of the disease. The strong hypoglycemic effects of this natural agent were probably due to its pancreatic actions, which are confirmed by elevation of serum insulin level, improving β-cell function and islet destruction and/or from extra pancreatic actions which was clear from decreasing insulin resistance and increasing glucose utilization by the skeletal muscles.

The demonstrated anti-hyperlipidemic effect of MC may be at least due to increasing insulin secretion which inhibits lipoprotein lipase and prevents lipolysis. The present investigation also suggested that this functional food has strong antioxidant activity and can play a protective role in reducing oxidative stress accompanied by DM and its complications.
